# Mindfulness, Depression, and Fall Risk in Low-Income Older Adults

**DOI:** 10.53520/rdhs2025.104164

**Published:** 2025-10-08

**Authors:** Aleeyah Uddeen, Janet Lopez, Renata Komalasari, Manika Petcharat, Veronica Decker, Tatiana Orozco, Ladda Thiamwong

**Affiliations:** 1 College of Nursing, University of Central Florida, Orlando, FL, USA; 2 Akademi Keperawatan Andalusia Jakarta, Indonesia; 3 Tzu Chi Hospital Indonesia; 4 Christine E. Lynn College of Nursing, Florida Atlantic University, Boca Raton, FL, USA.; 5 VA North Florida/South Georgia Veterans Health Syst em, Gainesville, FL, USA; 6 Disability, Aging, and Technology Cluster, University of Central Florida, Orlando, FL, USA

**Keywords:** Fall Prevention, Aging, Mental Well-being

## Abstract

**Introduction:**

Mindfulness, through mind-body practices, promotes awareness and presence. Its practice, along with reduced depression, may improve balance and lower fall risk in older adults. This study examined the association between mindfulness, depression, and fall risk among low-income, community-dwelling older adults.

**Methods:**

A descriptive cross-sectional study was conducted with 103 community-dwelling, low-income older adults, including 16 males and 87 females, with a mean age = 75.70 years (95% CI ± 7.19). Mindfulness was assessed with the Mindful Attention Awareness Scale (MAAS), depression using the Patient Health Questionnaire (PHQ-9) and fall risk with the CDC’s checklist for elderly accidents and injuries. Multiple regression analysis was conducted.

**Results:**

Mindfulness was negatively correlated with fall risk (*r*=−.437, *p*<.001), while depression was positively correlated with fall risk (*r*=.486, *p*<.001). These variables explained 31.1% of the variance in fall risk *(R*^*2*^ = .311, *p* < .001). Mindfulness (*β* = −.262, p =.006) and depression (*β* = .306, p =.001), respectively, were significantly associated with fall risk.

**Conclusions:**

This study demonstrates that mindfulness and depression are predictors of fall risk. Community-dwelling low-income older adults may benefit from mind-body exercises. These exercises can help them become more aware and enhance their mental health, which may lower their risk of falling.

## Introduction

The risk of falling among older adults is a significant issue, contributing to many injury-related deaths.^1,2^ Over one in four older adults experience a fall each year, and the likelihood of falling, and suffering related complications, increases with age.^3^ These falls can cause serious injuries and affect both physical and mental health, as many older adults experience multimorbidity linked to poor mental health, including depression.^4^

Mental health challenges like depression and anxiety can impair daily activities and awareness of surroundings, increasing fall risk.^5^ Depressive symptoms, such as cognitive impairment and low energy, elevate the likelihood of falls. Given the growing interest in holistic approaches to fall prevention,^6–8^ it is important to distinguish between mind-body practices, which integrate physical movement with mental focus (e.g., yoga, tai chi), and mindfulness, which refers more specifically to the psychological process of maintaining moment-to-moment awareness without judgment.^9^

Mindfulness, while often a component of mind-body practices, can also be cultivated independently through techniques such as meditation and focused breathing. These practices have shown promise in improving mental clarity, emotional regulation, and physical stability, which may help reduce fall risk.^10,11^ Research shows a bidirectional link between depression and fall risk.^12^ Symptoms such as slow gait and diminished mental processing speed increase the likelihood of falls.^13^ Cognitive impairment and poor balance also correlate with depressive symptoms, making older adults less aware of their surroundings.^14^

Mindfulness may offer a solution by enhancing awareness and reducing distractions. Defined as being present and attentive,^15^ mindfulness practices can be easily incorporated into daily routines without the need for complex equipment. Studies have shown that participating in mindfulness programs can lead to better balance and increased confidence in preventing falls.^7^

Older adults in low-income settings often engage less in physical activity,^16,17^ which further increases their risk of falling due to sedentary lifestyles.^18^ Additional factors like low education and inadequate housing also contribute to their fall risk^19^ and negatively impact overall health.^20^ While previous studies have examined fall risk and its physical determinants,^21^ limited research has explored the psychological dimensions^22^, particularly the roles of depression and mindfulness, in this context. Evidence suggests that depression may heighten fall risk,^12,14^ while mindfulness could serve as a protective factor by enhancing awareness and emotional regulation.^23^ However, these relationships remain underexplored among low-income older adults. Therefore, understanding the relationship between depression, mindfulness, and fall risk is crucial for addressing health disparities in this population. This study aims to examine the correlation between mindfulness, depression, and fall risk among community-dwelling older adults in low-income settings, emphasizing the importance of these factors in promoting better health outcomes.

## Scientific Methods

A descriptive cross-sectional survey was conducted to examine associations among mindfulness, depression, and fall risk in low-income older adults, controlling for age and gender due to their known influence on fall risk.^24^ This study is part of a larger cluster randomized controlled trial study focused on integrating technology-based body and mind interventions to prevent falls and address health disparities among low-income, community-dwelling older adults.^25^ It was approved by the University of Central Florida IRB (STUDY00003206) and pre-registered on ClinicalTrials.gov (NCT05778604).

### Participants and Sampling

We recruited 103 community-dwelling older adults from senior independent living apartments in Central Florida through newsletters, flyers, and word of mouth. Eligibility criteria included: (a) community-dwelling adults aged 60 or older; (b) residing in low-income settings in Orlando, Florida, as defined by the 2019 U.S. Census poverty thresholds for family households^26^; (c) no significant cognitive impairment, defined as a score ≥5 on the Memory Impairment Screen (MIS)^27^; and (d) residence in independent living facilities or private homes. All participants provided informed consent after being briefed on the study’s risks and benefits. A priori power analysis using G*Power 3.1.2 determined that a sample of 103 was sufficient to detect a medium effect size with two predictors (mindfulness and depression),^28^ assuming α = .05 and power = .80.

### Procedures

Trained research assistants met with the participants at the data collection site and explained the study objectives and procedures. After obtaining consent from the participants, they were given directions to complete questionnaires about mindfulness, depression, and fall risk. These surveys were either done in-person or online depending on their preference. For the online survey, a URL code that linked our survey was created and sent out to the participants. Screening and data collection were obtained on site in a common area at participants’ apartments or at senior community centers.

### Measures

#### Demographics:

Participants completed a background questionnaire that collected information on gender, age, education level, and perceived financial stability.

#### Mindfulness:

Mindfulness was measured using the Mindful Attention Awareness Scale (MAAS), which measures dispositional mindfulness, specifically the attentive awareness of present moment experience.^29^ This scale consists of fifteen-items, with lower scores indicating less awareness. The survey consists of a series of statements about everyday experiences and uses a 6-point Likert-type response scale ranging from 1 (almost always) to 6 (almost never). This scale has a Cronbach’s alpha of 0.89–0.93 and has shown convergent and discriminant validity.^30^

#### Depression:

Depression was assessed using the Patient Health Questionnaire-9 (PHQ-9) with lower scores indicting fewer depressive symptoms. A total of 10 items (e.g., feeling tired) on a 4-point scale, measuring symptoms of depression within the past 2 weeks, with a total score range of 0–27.^31^ This scale has a Cronbach’s alpha of 0.89.^31^

#### Fall Risk:

Fall risk was assessed using the CDC’s Stopping Elderly Accidents, Deaths, and Injuries (STEADI) fall risk checklist. The tool included 12 yes-or-no statements addressing physical and psychological factors related to falling. A score of 4 or more indicated an increased risk of falling.^32^ The checklist demonstrated a sensitivity of 73% to 80% in identifying current and future fall risk among community-dwelling older adults.^33^

### Statistical Analysis

This study examined the association between fall risk and mindfulness and depression, controlling age and gender. After data screening and cleaning, multiple regression analysis was conducted using the Statistical Package for Social Sciences (SPSS) version 28, with an alpha of .05 considered statistically significant. The assumptions of a standard multiple regression were assessed, including multicollinearity, singularity, outliers, normality, linearity, and homoscedasticity. Multicollinearity was examined by the correlation between variables (range= −.035 to .486) and the tolerance and variance inflation factor (VIF) fell within the acceptable value. Each independent variable is not a combination of others; therefore, there was no violation of singularity. According to the standardized residual plot, all values are between −3.0 and 3.0, with no violation for outliers. Residual scatterplots were used to assess the normality, linearity, and homoscedasticity; no violation of these assumptions was found.

## Results

### Sample characteristics

In total, 103 participants were involved in the statistical analysis (16 (15.5%) males, 87 (84.5%) females; mean age = 75.70, 95% CI ±7.19). Across the study sample, 73 (70.9%) were White, 72 (69.9%) had attained a college education or above, 49 (47.6%) perceived to earn more than enough financially despite living in low-income households. (See Table 1)

### Bivariate correlation of main variables

The mean and standard deviation (SD) and correlation coefficient of each variable are shown in Table 2. Fall risk was negatively correlated with mindfulness (*r*=−.437, p<.001), but positively correlated with depression (*r*=.486, p<.001). Mindfulness was negatively correlated with depression (*r*=−.461 p<.001). Age showed a positive correlation with fall risk (*r* = .241, p < .05), while its associations with mindfulness and depression were not statistically significant. Gender was not significantly correlated with any of the primary variables, indicating no meaningful differences in fall risk, mindfulness, or depression based on gender in this sample. (See Table 2)

### The correlation between mindfulness, depression, and fall risk

The results of the multiple regression analysis indicated that the model explained 31.1% of the variance (*R*^*2*^=.311, F (4,98) =11.05, *p* <.001). In other words, mindfulness and depression could reciprocally predict fall risk at 31.1%. It was found that the mindfulness scores (*β* = −.262, p =.006) and depressive symptoms (*β* = .306, p =.001) were statistically significantly predictive of STEADI scores, controlling age and gender. Findings of this study suggested that among low-income older adults, as depressive symptoms also increased, fall risk increased. As mindfulness decreased, fall risk increased.

Squared structure coefficients indicated that mindfulness contributed 68.7% ($r_{s}^{2}=.687$) to the majority of the obtained effect in this model, while depression contributed a slightly higher effect ($r_{s}^{2}=.725$ or 72.5%), suggesting depressive symptoms had a stronger effect than mindfulness in predicting fall risk, controlling age and gender. (See Table 3).

## Discussion

The aim of our study was to examine the correlation between mindfulness, depression, and fall risk among community-dwelling older adults in low-income settings.^34^ Our findings indicated that depression is strong predicator to fall risk, people with more symptoms of depression were more likely to be at risk of falling. Mindfulness is also an important predictor, those who were less mindful had a higher chance of falling. Overall, depression and mindfulness were the most important factors in predicting who might be at risk of falling.

Evidence shows that low-income older adults are at an increased risk of experiencing depression.^34^ Research has shown that depressive symptoms are a significant predictor of falls.^35,36,37^ Depression tends to diminish attention and awareness, particularly in older adults who may already struggle with cognitive decline.^38,39^ Elevated levels of depressive symptoms may increase the likelihood of falls due to reduced attention to surroundings. Kose et al.^37^ identified significant correlations between cognitive functioning, mobility, and depression, suggesting that impairments in these areas collectively contribute to fall risk. Therefore, it is important to address depression as part of a fall prevention strategy for low-income older adults.

Our findings show that higher levels of mindfulness are associated with lower levels of depression. This aligns with existing evidence that mindfulness-based interventions can effectively reduce depressive symptoms.^40,41^ Since depression is a predictor of fall risk, promoting mindfulness may not only support mental well-being but also contribute to fall prevention among older adults. Practicing mindfulness offers a simple method for community-dwelling older adults to enhance their cognitive functioning and mental well-being.^42,43^ Improved cognition can lead to greater awareness, thereby diminishing fall risk.^44,45^

Moreover, research supports that mindfulness can be an effective strategy to improve balance and thereby reduce fall risk in older adults.^46^ In our study, dispositional mindfulness was assessed using the Mindful Attention Awareness Scale (MAAS), which evaluates the ability to pay intentional, non-judgmental attention. Sardeli et al.^44^ found a significant correlation between dispositional mindfulness and balance capabilities, indicating that fostering mindfulness might enhance balance control among older adults. Although their study was a randomized controlled trial with a small sample of 48 older adults, it highlighted that a combination of mindful practices and exercise can improve balance control and potentially lower fall risk.^46^ Furthermore, another study found that mindfulness-based interventions can effectively reduce stress, enhance self-efficacy, and help manage both psychological and physical ailments among low-income older populations.^47^ These findings underscore the importance of incorporating such interventions as part of fall prevention strategies

Future implications may involve healthcare providers incorporating mindfulness and mental health strategies to mitigate fall risk among older adults. Potential strategies could include breathing exercises and meditation programs. Additionally, implementing mindfulness interventions or depression awareness programs in low-income communities could proactively address fall risks. Community-based programs, such as tai chi and yoga, which integrate physical exercise with mindfulness practices, may also help in reducing fall incidents.

### Limitations

This study’s cross-sectional design limits the ability to examine how mindfulness, depression, and fall risk change over time. Therefore, a longitudinal or experimental study on these variables should be conducted for further investigation. Another limitation is that there are additional factors associated with fall risks among older adults that need to be evaluated in future studies, such as comorbidities. Certain minority groups were not well represented such as African Americans, Asian American, Pacific Islanders, or Native Americans. Furthermore, the selection of participants was not randomized, had a small sample size, and 85.5% were female. Thus, these results cannot be generalized to other populations. Cohort differences across age and gender groups were not specifically examined. While age and gender were controlled as covariates, the sample size may have limited the ability to detect age-specific differences. Future studies with larger samples could explore these variations to better understand how mindfulness, depression, and fall risk differ across older adult subgroups.

## Conclusions

This study revealed that mindfulness and depression were correlated with fall risk with depressive symptoms being a stronger predictor. Additionally, mindfulness was negatively correlated with depression. To our knowledge, this research represents the first investigation of the relationship between mindfulness, depression, and fall risk, highlighting the need for further studies in this area. Future research could examine how changes in mindfulness and depression over time are associated with fall risk within low-income settings or across diverse demographics in a longitudinal study. Older adults in low-income communities may benefit from mindfulness practices and mental health support to reduce fall risks. To enhance safety and well-being among older adults living in the community, future fall risk intervention programs should incorporate mindfulness techniques and provide mental health support.

## Figures and Tables

**Table 1.** Participant characteristics (N=103).

Variables		Mean	SD
Age		75.7	7.19
		Frequency	Percentage (%)
Gender	Female	87	84.5
	Male	16	15.5
Education	Lower than high school	2	1.9
	High school	29	28.2
	College or above	72	69.9
Financial Situation	Much less than adequate	2	1.9
	Less than adequate	7	6.8
	Just enough	37	35.9
	More than adequate	49	47.6
	Much more than adequate	8	7.8
Race/Ethnicity	White	73	70.9
	African American	8	7.8
	Hispanic	20	19.3
	Asian	2	1.9
Age cohort	60–69 years old	21	20.4
	70–79 years old	54	52.4
	80 and over	28	27.2

**Table 2.** Spearman correlation coefficients of variables among participants (N=103).

Variable	M ± SD	M±(P25,27)	1.	2.	3.	4.	5.
1. Fall risk	2.5±2.62		1				
2. Mindfulness	77.02±11.11		−.437**	1			
3. Depression	2.13±2.41		.486**	−.461**	1		
4. Age	75.70±1.84		.241*	.003	−.035	1	
5. Gender	-	2(2,2)	.119	.057	.086	−.107	1

** p < .001,

* p < .05;

M±SD: mean ±Standard Deviation; 1, 2, 3, 4 follows normal distribution and is described as M±SD; 5 does not follow normal distribution and is described as M±(P25,27)

**Table 3.** Beta weights and structure coefficient for STEADI scores (*N*= 103).

Variable	B	*β*	t	*p*	*r*_*s*_	$r_{s}^{2}$
1. Mindfulness	−.062	−.262	−2.82	.006	−.829	.687
2. Depression	.333	.306	3.30	.001	.852	.725
3. Gender	1.36	.189	2.24	.028	.022	<.01
4. Age	.085	.232	2.75	.007	−.002	<.01

* *Note*. All beta weights were statistically significant at *p*<.05. *r*_*s*_ = structure coefficient.

## References

[1] Prevention. CfDCa. Older Adult Falls Data. Centers for Disease Control and Prevention. Accessed August 21, 2025. https://www.cdc.gov/falls/data-research/index.html

[2] Rogers ME, Rogers NL, Takeshima N, Islam MM. Methods to assess and improve the physical parameters associated with fall risk in older adults. Prev Med. Mar 2003;36(3):255–64. doi:10.1016/s0091-7435(02)00028-2 12634016

[3] Aging NIo. Falls and Fractures in Older Adults: Causes and Prevention. National Institutes of Health. Updated September 12, 2022. 2025. https://www.nia.nih.gov/health/falls-and-falls-prevention/falls-and-fractures-older-adults-causes-and-prevention

[4] Asante D, Rio J, Stanaway F, Worley P, Isaac V. Psychological distress, multimorbidity and health services among older adults in rural South Australia. J Affect Disord. Jul 15 2022;309:453–460. doi:10.1016/j.jad.2022.04.140 35490879

[5] Lohman MC, Mezuk B, Fairchild AJ, Resciniti NV, Merchant AT. The role of frailty in the association between depression and fall risk among older adults. Aging Ment Health. Sep 2022;26(9):1805–1812. doi:10.1080/13607863.2021.1950616 35993919 PMC9395731

[6] Loewenthal J, Berning MJ, Wayne PM, Eckstrom E, Orkaby AR. Holistic frailty prevention: The promise of movement-based mind-body therapies. Aging Cell. Jan 2024;23(1):e13986. doi:10.1111/acel.13986 37698149 PMC10776124

[7] Parra DC, Wetherell JL, Van Zandt A, Brownson RC, Abhishek J, Lenze EJ. A qualitative study of older adults’ perspectives on initiating exercise and mindfulness practice. BMC Geriatr. Dec 23 2019;19(1):354. doi:10.1186/s12877-019-1375-9 31865906 PMC6927182

[8] Sherrington C, Fairhall NJ, Wallbank GK, Exercise for preventing falls in older people living in the community. Cochrane Database Syst Rev. Jan 31 2019;1(1):Cd012424. doi:10.1002/14651858.CD012424.pub2 30703272 PMC6360922

[9] Kabat-Zinn J. Mindfulness-based interventions in context: past, present, and future. 2003;

[10] Chang MY, Lin CL, Wu TM, Chu MC, Huang TH, Chen HY. Eight forms of moving meditation for preventing falls in community-dwelling middle-aged and older adults. Forsch Komplementmed. 2013;20(5):345–52. doi:10.1159/000355842 24200824

[11] Li F, Harmer P, Fisher KJ, Tai Chi and fall reductions in older adults: a randomized controlled trial. J Gerontol A Biol Sci Med Sci. Feb 2005;60(2):187–94. doi:10.1093/gerona/60.2.187 15814861

[12] Iaboni A, Flint AJ. The complex interplay of depression and falls in older adults: a clinical review. Am J Geriatr Psychiatry. May 2013;21(5):484–92. doi:10.1016/j.jagp.2013.01.008 23570891 PMC4880473

[13] Kvelde T, McVeigh C, Toson B, Depressive symptomatology as a risk factor for falls in older people: systematic review and meta-analysis. J Am Geriatr Soc. May 2013;61(5):694–706. doi:10.1111/jgs.12209 23617614

[14] Gambaro E, Gramaglia C, Azzolina D, Campani D, Molin AD, Zeppegno P. The complex associations between late life depression, fear of falling and risk of falls. A systematic review and meta-analysis. Ageing Res Rev. Jan 2022;73:101532. doi:10.1016/j.arr.2021.101532 34844015

[15] Kabataş Yıldız M, Orak OS. The effect of the Mindfulness-Based Stress Reduction program on the level of perceived stress and geriatric depression in older adults: a randomised controlled study. Psychogeriatrics. Mar 2023;23(2):261–272. doi:10.1111/psyg.12929 36594217

[16] Devereux-Fitzgerald A, Powell R, French DP. The Acceptability of Physical Activity to Older Adults Living in Lower Socioeconomic Status Areas: A Multi-Perspective Study. Int J Environ Res Public Health. Nov 10 2021;18(22)doi:10.3390/ijerph182211784 PMC8619977 34831546

[17] Annear MJ, Cushman G, Gidlow B. Leisure time physical activity differences among older adults from diverse socioeconomic neighborhoods. Health Place. Jun 2009;15(2):482–490. doi:10.1016/j.healthplace.2008.09.005 19038571

[18] Jiang Y, Wang M, Liu S, Ya X, Duan G, Wang Z. The association between sedentary behavior and falls in older adults: A systematic review and meta-analysis. Front Public Health. 2022;10:1019551. doi:10.3389/fpubh.2022.1019551 36438277 PMC9691853

[19] Choi SD, Andrade EL. Multiple-Level Fall Risks Associated with Socioeconomic Factors Among Community-Dwelling Older Adults: A Systematic Review. 2022:10–27.

[20] Brady D, Kohler U, Zheng H. Novel Estimates of Mortality Associated With Poverty in the US. JAMA Intern Med. Jun 1 2023;183(6):618–619. doi:10.1001/jamainternmed.2023.0276 37067817 PMC10111231

[21] Maruszewska A, Ambroży T, Rydzik Ł. Risk factors and socioeconomic determinants of falls among older adults. Frontiers in Public Health. 2025;13:1571312. 40182529 10.3389/fpubh.2025.1571312 PMC11966436

[22] Namoos A, Thomson N, Olson C, Aboutanos M. Physical Injury and Psychological Impact: Understanding the High Risk of Depression on Older Adults with Recurrent Falls. Advances in Geriatric Medicine and Research. 2024;6(4):e240008. e240008. doi:10.20900/agmr20240008 40787151 PMC12333890

[23] Hoang P, Moore K, Kwan M. Examining the Feasibility of a Mindfulness Intervention for the Prevention of Falls: A Pilot Study. Can J Aging. Dec 2020;39(4):626–633. doi:10.1017/S0714980820000033 32248857

[24] Kakara R, Bergen G, Burns E. Understanding the association of older adult fall risk factors by age and sex through factor analysis. Journal of applied gerontology. 2023;42(7):1662–1671. 36724197 10.1177/07334648231154881 PMC10258133

[25] Thiamwong L, Xie R, Park J-H, Lighthall N, Loerzel V, Stout J. Optimizing a technology-based body and mind intervention to prevent falls and reduce health disparities in low-income populations: protocol for a clustered randomized controlled trial. JMIR research protocols. 2023;12(1):e51899. 37788049 10.2196/51899 PMC10582821

[26] Poverty Thresholds. The United States Census Bureau. Accessed October 5, 2025 https://www.census.gov/data/tables/time-series/demo/income-poverty/historical-poverty-thresholds.html

[27] Folstein MF, Folstein SE, McHugh PR. “Mini-mental state”. A practical method for grading the cognitive state of patients for the clinician. J Psychiatr Res. Nov 1975;12(3):189–98. doi:10.1016/0022-3956(75)90026-6 1202204

[28] Faul F, Erdfelder E, Buchner A, Lang AG. Statistical power analyses using G*Power 3.1: tests for correlation and regression analyses. Behav Res Methods. Nov 2009;41(4):1149–60. doi:10.3758/BRM.41.4.1149 19897823

[29] Brown KW, Ryan RM. The benefits of being present: mindfulness and its role in psychological well-being. Journal of personality and social psychology. 2003;84(4):822. 12703651 10.1037/0022-3514.84.4.822

[30] Black DS, Sussman S, Johnson CA, Milam J. Psychometric assessment of the Mindful Attention Awareness Scale (MAAS) among Chinese adolescents. Assessment. Mar 2012;19(1):42–52. doi:10.1177/1073191111415365 21816857 PMC3705937

[31] Kroenke K, Spitzer RL, Williams JB. The PHQ-9: validity of a brief depression severity measure. Journal of general internal medicine. 2001;16(9):606–613. 11556941 10.1046/j.1525-1497.2001.016009606.x PMC1495268

[32] Rubenstein LZ, Vivrette R, Harker JO, Stevens JA, Kramer BJ. Validating an evidence-based, self-rated fall risk questionnaire (FRQ) for older adults. Journal of safety research. 2011;42(6):493–499. 22152267 10.1016/j.jsr.2011.08.006

[33] Nithman RW, Vincenzo JL. How steady is the STEADI? Inferential analysis of the CDC fall risk toolkit. Arch Gerontol Geriatr. Jul-Aug 2019;83:185–194. doi:10.1016/j.archger.2019.02.018 31075677

[34] Xue Y, Lu J, Zheng X, The relationship between socioeconomic status and depression among the older adults: the mediating role of health promoting lifestyle. Journal of affective disorders. 2021;285:22–28. 33621711 10.1016/j.jad.2021.01.085

[35] Pereira MEA, Santos GS, Almeida CR, Association between Falls, Fear of Falling and Depressive Symptoms in Community-Dwelling Older Adults. Healthcare (Basel). Aug 16 2024;12(16)doi:10.3390/healthcare12161638 PMC11353576 39201196

[36] Kvelde T, McVeigh C, Toson B, Depressive symptomatology as a risk factor for falls in older people: systematic review and meta-analysis. Journal of the American Geriatrics Society. 2013;61(5):694–706. 23617614 10.1111/jgs.12209

[37] Kim JH, Song JH, Wee JH, Lee JW, Choi HG. Depressive Symptoms, Subjective Cognitive Decline, and Subjective Sleep Quality Are Associated with Slips and Falls: Data from the Community Health Survey in Korean Adults. Gerontology. 2022;68(5):518–528. doi:10.1159/000518007 35580570

[38] Potter GG. Depression and cognitive impairment in older adults. Psychiatric Times. 2007;24(13):23–23.

[39] Forbes M, Lotfaliany M, Mohebbi M, Depressive symptoms and cognitive decline in older adults. International psychogeriatrics. 2024;36(11):1039–1050. 38623851 10.1017/S1041610224000541

[40] Reangsing C, Rittiwong T, Schneider JK. Effects of mindfulness meditation interventions on depression in older adults: A meta-analysis. Aging Ment Health. Jul 2021;25(7):1181–1190. doi:10.1080/13607863.2020.1793901 32666805

[41] Hofmann SG, Gómez AF. Mindfulness-Based Interventions for Anxiety and Depression. Psychiatr Clin North Am. Dec 2017;40(4):739–749. doi:10.1016/j.psc.2017.08.008 29080597 PMC5679245

[42] Fiocco AJ, Mallya S. The importance of cultivating mindfulness for cognitive and emotional well-being in late life. Journal of evidence-based complementary & alternative medicine. 2015;20(1):35–40. 25331094 10.1177/2156587214553940

[43] Lee EKP, Wong B, Chan PHS, Effectiveness of a mindfulness intervention for older adults to improve emotional well-being and cognitive function in a Chinese population: A randomized waitlist-controlled trial. International Journal of Geriatric Psychiatry. 2022;37(1) 10.1002/gps.5616 34415638

[44] Robinson JE, Kiely J. Preventing falls in older adults: can improving cognitive capacity help? Cogent Psychology. 2017;4(1):1405866.

[45] Segev-Jacubovski O, Herman T, Yogev-Seligmann G, Mirelman A, Giladi N, Hausdorff JM. The interplay between gait, falls and cognition: can cognitive therapy reduce fall risk? Expert review of neurotherapeutics. 2011;11(7):1057–1075. 21721921 10.1586/ern.11.69 PMC3163836

[46] Sardeli AV, Sartori CR, Santos WM, de Freitas Brandão A, Chacon-Mikahil MPT. Dispositional mindfulness influences the balance control in elderly. Journal of Innovation and Healthcare Management. 2019;2:1–9.

[47] Szanton SL, Wenzel J, Connolly AB, Piferi RL. Examining mindfulness-based stress reduction: perceptions from minority older adults residing in a low-income housing facility. BMC Complement Altern Med. May 31 2011;11:44. doi:10.1186/1472-6882-11-44 21627807 PMC3123255

